# Implicit theories concerning the intelligence of individuals with Down syndrome

**DOI:** 10.1371/journal.pone.0188513

**Published:** 2017-11-22

**Authors:** Claire Enea-Drapeau, Michèle Carlier, Pascal Huguet

**Affiliations:** 1 PSYCLE, Aix Marseille Univ, Aix-en-Provence, France; 2 LPC, Aix Marseille Univ, CNRS, Marseille, France; 3 Université Clermont Auvergne et CNRS, LAPSCO, Clermont-Ferrand, France; TNO, NETHERLANDS

## Abstract

Studies over the past three decades have shown that learning difficulties are not only determined by neurological disorders, but also by motivational and/or socio-cognitive factors Among these factors, implicit theories of intelligence (also referred to as conceptions, mindsets or beliefs about intelligence) are key elements. The belief that intelligence is fixed (entity theory), as opposed to malleable (incremental theory), is generally associated with negative teaching practices and poorer student outcomes, yet beliefs about the intelligence of individuals with intellectual disabilities have not received much attention. We propose the first study on conceptions of intelligence of persons with intellectual disabilities, here people with Down syndrome. Participants were 55 professionally qualified people working with individuals with intellectual disabilities and 81 adults from the community. We compared what both groups of participants believe about intelligence of typical people and what they believe about the intelligence of individuals with Down syndrome. We also investigated implicit theories of intelligence as predictors of explicit judgments about intelligence and implicit attitudes toward people with Down syndrome. Whatever the work experience in the field of intellectual disability, implicit theories of intelligence were found to be less incremental when considering people with Down syndrome than when considering typical people; and the stronger the belief in entity theory, the more negative (and less positive) the judgments expressed explicitly. Implicit theories of intelligence were also found to be predictors of negative implicit attitude but only in adults from the community. These findings offer prospects for improving practices by people working in the field of intellectual disability. They might interest a wide range of people caring for people with intellectual disabilities, such as teachers, but also other professional caregivers, and other scientists focusing on intellectual disabilities or social cognition.

## Introduction

The main phenotypic characteristics of Down syndrome (DS) include facial features and intellectual disability. DS is caused by a chromosomal aberration, trisomy 21. This trisomy is the most frequent genetic disorder associated with intellectual disabilities and learning difficulties [1]. Despite their intellectual disability, people with DS are characterized by large variability in cognitive traits[2–4] and cognitive modifiability in people with DS has been shown [5,6]. Most children and young people with DS receive individualized supports related to their special needs and dealing with their difficulties requires attention from educational professionals and researchers [7–9]. Of particular interest here, studies over the past three decades have shown that learning difficulties are not only determined by neurological disorders, but also by motivational and/or socio-cognitive factors [10,11]. Among these factors, implicit theories of intelligence (also referred to as conceptions, mindsets or beliefs about intelligence; see [12–14] are key elements. The word intelligence is largely used in various settings including professional contexts, yet it is associated with different personal conceptions of intelligence. Therefore, it is important to investigate beliefs on intelligence especially in people working on skills development. Dweck and colleagues distinguished between entity theorists, that is, people who consider that intelligence is fixed and unchangeable (fixed mindset), and incremental theorists which are people who consider that intelligence may result from hard work and effort, and is therefore malleable to a certain extent (growth mindset) [12–14]. Research in this area revealed that beliefs in fixed intelligence are generally associated with negative teaching practices and lower student outcomes [15–21]. Leroy et al., for example, found that teachers who held a theory of fixed academic ability displayed less support for autonomy in the classroom, while an autonomy climate generally fosters student intrinsic motivation [16]. Compared to teachers with an incremental theory, those with a fixed theory are also more likely to judge student’s abilities on the basis of first impressions and initial outcomes [20,22]. Teachers’ implicit theories of intelligence were also found to influence their feedback to students. Teachers believing that intelligence is fixed are more likely to unknowingly adopt teaching practices that undermine motivation when working with low achievement students. They may also use “kind” strategies for these students, for example by assigning less homework, which yet could have detrimental effects on their academic achievement. Such feedback proved also detrimental to students’ motivation and expectations for their own performance [20]. In many studies, Dweck and colleagues have shown that praise as expressed by teachers, depending on whether they laud the mastery of a given task or internal dispositions (intelligence and abilities), will either promote heightened motivation and efficient learning or create self-defeating behavior [12,23–25]. In short, whereas the use of feedback emphasizing internal dispositions generally enhances a fixed conception of intelligence in students, leading to less efficient learning strategies and lower achievement, feedback emphasizing hard work and effort are more likely to promote an incremental conception and produce higher levels of achievement [12].

Studies have reported that entity theorists, compared to incremental theorists, tend to make more rapid and stereotypical judgments [26–29]. Because of their facial features (e.g., round face and oblique palpebral fissures), people with DS are readily identified as such, and as the DS condition is associated with stereotypes, the people concerned are therefore likely to be stigmatized [30]. Surprisingly, the question of whether people make use of similar or different conceptions of intelligence about people with intellectual disabilities, compared to the non-disabled population, has been largely overlooked. Given the importance that these conceptions may ultimately have on learning, this question merits special attention. To fill this gap, the present study examined beliefs about the intelligence of individuals with or without DS. We compared beliefs of participants working with people with intellectual disabilities (hereafter referred to as “professionals”) to beliefs of participants not working with people with intellectual disabilities (hereafter referred to as “non-professionals”). We also investigated the relationship between participants’ implicit theories of intelligence and their explicit (overt) judgments as well as implicit (presumably unconscious) attitudes toward people with DS.

It was expected that implicit theories of intelligence when judging individuals with DS would be less incremental than when judging people without DS. Many people have a naïve conception of the relationships between genes and behavior, which does not reflect the state of the scientific knowledge [31]. As trisomy 21 is a genetic (chromosomal) disorder, people with DS are more likely to be associated with features characterized as innate and impossible to change. We also expected an effect of work experience in the field of intellectual disability, the beliefs about the intelligence of people with DS would be more incremental in professionals than in non-professionals, simply because the former usually focus on skills improvement. Finally, we expected that the stronger the belief in entity theory, the more likely the people would be to make stereotypical judgments about intelligence and to show negative implicit attitude.

## Material and methods

### Participants

Participants were 136 adults, including 55 people working with people with intellectual disabilities (such as individuals with DS). These 55 participants (the “professionals”) were the caregivers who participated in Enea-Drapeau, Carlier and Huguet’s study [30]. They were recruited in institutions for people with intellectual disabilities, and in mainstream schools if they were working in classrooms for students with special needs. It was a mixed group including caregivers (special education teachers, speech therapists, nurses, social workers, etc.). Psychologists and medical doctors were not recruited as they would have been more aware than other people of effects of beliefs and stereotyping concerning intellectual disability. All these professionals had work experience with individuals with DS. They were 44 women and 11 men (mean age = 39.1 years, SD = 9.33, range: 23–62; mean years of formal education = 13.7, SD = 1.68, range: 11–16). The remaining 81 participants (the “non-professionals”) included the 55 non-student adults who participated in Enea-Drapeau et al.’s study [30]. They did not have work experience in the field of intellectual disability, and were recruited from the general population via adverts for participants in research about DS. They were 47 women and 34 men (mean age = 36.05 years, SD = 14.35, range: 18–67; mean years of formal education = 14.14, SD = 2.05 range: 11–17). Twenty-three non-professionals declared that they have known an individual with DS in their personal environment.

### Procedure

All participants gave written informed consent to participate in the study, presented as a study on the “face perception of people with trisomy 21” (The term “syndrome de Down” (DS) is very rarely used in France. People with DS are known as “people with trisomy 21”). Each participant was seated individually in a room and asked to perform tasks on a computer. The project obtained approval from the Ethics Committee of Aix-Marseille Univ. (Avis Carlier 18.11.09).

#### Implicit theories of intelligence

The implicit theories of intelligence were assessed using two scales: the Dweck’s 8-item Theories of Intelligence Scale measuring what participants believe about intelligence in general (TIS, [12]—referred to as “TIS-General”). The scale contains 4 entity (or fixed) items and 4 malleable (or growth) items. The French version of the 8-item scale was made with the collaboration of two native English speakers (back-translation method). The second scale was an adaptation for measuring what people believe about the intelligence of individuals with DS (referred to as “TIS-DS”). Entity items such as “People have a certain amount of intelligence, and people can’t really do much to change it” in TIS-General became “People with trisomy 21 have a certain amount of intelligence, and people with trisomy 21 can’t really do much to change it” in TIS-DS. Likewise malleable items such as “People can always substantially change how intelligent they are” (TIS-General) became “People with trisomy 21 can always substantially change how intelligent they are” (TIS-DS). Participants indicated whether they agreed or disagreed on each item (presented in random order) using a Likert-type scale (ranging from 1: “strongly disagree” to 6: “strongly agree”). The order of the two TISs was counterbalanced across participants.

The four malleable items were reverse-coded so that the eight items measured entity theory [32]. TIS-General and TIS-DS scores were thus obtained by averaging the 8 items of each scale. Internal consistencies for the two TIS scores were very high in each group of participants (Cronbach’s αs: non-professionals: .92 for TIS-General and TIS-DS; Professionals: .88 and .91 for TIS-General and TIS-DS respectively). The two TIS scores correlated significantly in each group of participants (*r*_Professional_ = 0.49; *r*_Non-professional_ = 0.58; *ps* ≤ .001). These correlations were not very high supporting the assumption that the two scales targeted two different conceptions of intelligence.

#### Explicit judgments

We followed the procedure described by [30]. Participants made explicit evaluations of 12 children with full trisomy 21 (6 girls and 6 boys) seen as portrait photographs presented in a random order at the center of the computer screen along with a word (bottom of screen). The photographs were standardized, showing only the face, with a neutral expression, against a blue background. Participants evaluated the pictures for 3 traits related to intelligence (“éducable” [educable], “intelligent” [intelligent] and “bête” [stupid]), which were included in a list of 12 traits used in the implicit association test (see below). Participants had to indicate spontaneously to what extent each of the 12 traits matched the picture, using a Likert-type scale ranging from 1: “strongly disagree” to 6: “strongly agree”. Each trait was scored as the mean of all scores for all 12 pictures of faces with DS.

#### Implicit attitudes

Implicit attitudes to people with DS were assessed using an implicit association test [33], a well-known technique whereby response latency is used to capture the relative strength with which some groups of people are associated with positive versus negative attributes in memory [30,34,35]. Participants classified two types of stimuli displayed on the computer screen: 18 children's faces and 12 personality traits, using two designated keyboard keys. Children’s faces were 6 photographed faces of typically developing children and the 12 photographed faces of children with full trisomy 21 described above. Personality traits were 6 positive traits (“humain” [human], “affectueux” [affectionate]”, “éducable” [educable], “attachant” [endearing], “sociable” [sociable/friendly], and “intelligent” [intelligent]), and 6 negative traits (“méchant” [mean], “bête” [stupid], “laid” [ugly] “gênant” [annoying], “agressif” [aggressive], and “déformé” [deformed]). Participants were told that a word or picture of a face would be displayed in the center of the screen and that they would have to choose which key to press as quickly as possible, classifying each stimulus (word or picture) in one of two categories (Positive vs. Negative for words, and DS vs. Normal for pictures). It was expected that people with a negative implicit attitude toward DS would have faster reaction times when faces of typically developing children and positive traits shared the same response key, and when the faces of children with DS and negative traits shared the other response key. In other words, we expected slower reaction times for the opposite combinations of stimuli: pictures of typically developing children and negative traits (with the same key), and pictures of children with DS and positive traits (with the other key). Implicit attitude score (*D* score) was calculated using the scoring algorithm recommended by [36]. It used a metric that was calibrated by each respondent’s latency variability and included a latency penalty for errors. Higher scores mean stronger negative implicit attitudes to children with DS.

Testing order was the same for all participants: first the IAT, then they expressed their explicit judgments, and finished the session with the two TISs.

### Statistical analyses

Proportions of participants viewing intelligence as incremental and fixed were compared with Chi^2^. Differences in theories of intelligence within and between the two groups of participants (Professionals *vs*. Non-professionals) were tested with an ANOVA. We explored the relationships between theories of intelligence (TIS-General and TIS-DS) on explicit judgments about intelligence and implicit attitudes using hierarchical multiple regression analyses. Two models of regression analysis were run for each dependent variable: trait scores for the three traits related to intelligence (“intelligent”, “stupid” and “educable”) and implicit attitude. The first model included only one predictor (TIS-General). In the second model, hierarchical regression tested TIS-DS independently of TIS-General.

## Results

### Implicit theories of intelligence

In non-professionals, 23 participants declared they knew personally an individual with DS. A mixed ANOVA design with knowing DS as independent variable and TIS as repeated measures was carried out to test the effect of this knowledge on TIS. The main effect of knowing DS was not significant and accounted for a very low percentage of variance (*F*_1,79_ = 0.031, p = 0.86, partial *η*^*2*^ < 0.001). TIS-General score was significantly lower (M = 2.82, SD = 1.01) than TIS-DS score (M = 3.03, SD = 1.02); *F*_1,79_ = 4.031, p *<* 0.05, partial *η*^*2*^ = 0.05). The interaction between knowing DS and TIS was not significant (*F*_1,79_ = 0.036, p = 0.85, partial *η*^*2*^ < 0.001). Therefore, we did not exclude these 23 participants from the group of non-professionals in the subsequent analyses.

No matter what group (work experience in the field of intellectual disability or not) or TIS, most participants viewed intelligence as incremental (the mean TIS score was below 3.5). On both TIS scores, there was no effect of work experience in the percentages of entity and incremental theorists (χs^2^ p > 0.50). Fewer participants (67.6%) considered intelligence to be incremental on TIS-DS compared to 77.9% on TIS-General (McNemar’s Test, χ^2^ = 5.28, p = 0.022).

A mixed ANOVA design with work experience in the field of intellectual disability as independent variable and TIS as repeated measures was carried out. The main effect of work experience was not significant (*F*_1,134_ = 0.36, p = 0.55, partial *η*^*2*^ = 0.003). TIS-General score was significantly lower (M = 2.75, SD = 0.96) than TIS-DS score (M = 3.03, SD = 0.93); *F*_1,134_ = 13.83, p < 0.001, partial *η*^*2*^ = 0.09). The interaction between work experience and TIS was not significant (*F*_1,134_ = 1.025, p = 0.31, partial *η*^*2*^ = 0.01). Mean scores on TIS-General and on TIS-DS were, for the professionals: 2.65 (SD = 0.86) and 3.03 (SD = 0.94), and for the non-professionals 2.81 (SD = 1.02) and 3.03 (SD = 0.93).

### Explicit judgments and implicit attitudes toward people with DS

Descriptive statistics on explicit judgments about intelligence and implicit attitudes of participants in the two groups and differences between the two groups (*t* test values and effect sizes—Cohen’s *d*) are presented in Table 1. Professionals were more positive compared to non-professionals, and considered people with DS to be more educable, more intelligent and less stupid compared to non-professionals. Both groups expressed negative implicit attitudes (mean IAT scores were significant, ps < 0.001); the IAT was smaller for professionals than for non-professionals. Effect sizes of the differences between the two groups ranged from medium to large.

**Table 1:** Descriptive statistics (Mean scores and standard deviation in parentheses) on explicit judgments and implicit attitude. *t-* tests and Size of the Difference Between the Two Groups of Participants (Cohen’s *d*).

	Non-professional	Professional	*t* (134)	Cohen’s *d*
Intelligent	3.62 (1.09)	4.28 (1.04)	-3.518^a^	0.608
Educable	4.42 (0.94)	5.19 (0.75)	-5.095^a^	0.880
Stupid	2.66 (1.02)	1.95 (0.89)	4.169^a^	0.720
Implicit attitude	0.59 (0.35)	0.35 (0.44)	3.502^a^	0.605

^a^p ≤ 0.001

Higher scores mean stronger attribution to trait and stronger negative implicit attitude.

### Theories of intelligence as predictors of explicit judgments and implicit attitudes

The summaries of the four hierarchical multiple regression analyses for each group are presented in Table 2. In six of the eight analyses, the best models were the model 2 where the two TISs were added. For professionals, TIS-DS predicted the score of two outcome variables “intelligent” and “stupid” after controlling the effect of TIS-General. With a more malleable conception of intelligence of people with DS, there was an increase in perceived intelligence and a decrease in perceived stupidity. For non-professionals, TIS-DS predicted the score of all outcome variables “intelligent”, “stupid”, “educable” and implicit attitude after controlling the effect of TIS-General. With a more malleable conception of intelligence, there was an increase in perceived intelligence and perceived educability, and a decrease in perceived stupidity and negative implicit attitude. For professionals, TIS-General was found to be the only predictor of “educable” (model 2 was non-significant), and no regression model fit the data for implicit attitude (see Table 2). The TIS scores explained higher percentage of variance in the non-professionals compared to the professionals (total R^2^ ranges between 12% and 35%, and between 3% and 28%, respectively, see Table 2). The percentage of explained variance was almost 1.5 times larger for “stupid” in the non-professionals compared to the professionals (0.316 *vs* 0.231). Implicit theories of intelligence explained more accurately judgments (are better predictors) in the non-professionals than in the professionals.

**Table 2:** Hierarchical multiple regression analyses predicting outcome variables from TIS-General and TIS-DS scores in the two groups of participants.

		Outcome variable															
		Intelligent				Stupid				Educable				Implicit attitude			
	Predictor	*Δ R*^2^	*p*	*β*	*p*	*Δ R*^2^	*p*	*β*	*p*	*Δ R*^2^	*p*	*β*	*p*	*Δ R*^2^	*p*	*β*	*p*
**Non Professional (n = 81)**																	
Model 1	TIS-General	0.037	0.086	-0.193	.086	0.039	0.078	0.197	0.078	0.010	0.382	-0.098	0.382	0.004	0.591	-0.061	0.591
Model 2	TIS-General	0.311	< 0.0001	0.197	0.084	0.277	< 0.0001	-0.175	0.130	0.283	< 0.0001	0.277	0.019	0.112	0.002	-0.297	0.026
	TIS-DS			-0.680	< 0.0001			0.645	< 0.0001			-0.652	< 0.0001			0.409	0.002
	*Total R*^2^	0.348	< 0.0001			0.316	< 0.0001			0.293	< 0.0001			0.116	0.008		
**Professional (n = 55)**																	
Model 1	TIS-General	0.187	0.001	-0.432	0.001	0.129	0.007	0.359	0.007	0.232	< 0.0001	-0.481	< 0.0001	0.003	0.702	0.053	0.702
Model 2	TIS-General	0.098	0.010	-0.258	0.060	0.103	0.011	0.18	0.200	0.039	0.099	-0.371	0.008	0.027	0.235	-0.039	0.806
	TIS-DS			-0.358	0.010			0.367	0.011			-0.227	0.099			0.188	0.235
	*Total R*^2^	.284	< .0001			.231	.001			.271	< .0001			.030	.457		

*Δ R*^2^ = increment of change in *R*^2^, *β* = standardized regression coefficient

## Discussion

Our study has a two-fold goal. Firstly, we examined what professionals working with people with intellectual disabilities and non-professionals believe about intelligence in general [12], and what they believe about the intelligence of individuals with DS. Secondly, we tested the effect of work experience in the field of intellectual disability on the beliefs about the intelligence of individuals with DS. Participants were less frequently classified as incremental theorists when considering the intelligence of people with DS compared to considering the intelligence of typical people: more than 77% of participants viewed people’s intelligence as incremental, but only 67% considered the intelligence of people with DS to be incremental. These findings do not tally with observations in the one published study on conceptions of intelligence of people with learning disabilities [37]. In this study, Gutshall investigated the impact of the learning disabilities label on teachers’ implicit views about the stability of student ability [37]. The author found no difference in the proportion of teachers with a growth/fixed mindset when considering the intelligence of people with or without learning disabilities. The difference between our findings and Gutshall’s findings may be explained by differences in experimental designs. Gutshall worked on learning disabilities, which is not necessarily linked to intellectual disabilities, and the assessment of the teachers’ conceptions of the intelligence of students was done from a descriptive written scenario of students struggling in school, without any details of any learning disability. In our study, not only were learning disabilities identified as a specific disability but they also were embodied by people with DS, caused by a chromosomal aberration (trisomy 21), i.e. a congenital disorder with effects usually perceived as being permanent. Therefore, social judgments tend to be more fixed, being related to permanent features. As expected, conceptions of intelligence of participants were less incremental when considering the intelligence of people with DS than when considering the intelligence of typical people. However contrary to our expectations, we did not observe significant difference in conceptions of intelligence according to work experience in the field of intellectual disability. We found that professionals, like ordinary people, can also hold a fixed theory of the intelligence about people in general and can be more fixed theorists concerning people with an intellectual disability such as DS than concerning people in general. Yet these are the professionals who are supposed to care for people with disabilities and help them develop their abilities. We observed a new aspect of the social perception of people with DS: whatever the work experience their intelligence was viewed as less incremental compared to intelligence of typical people. Professionals did not consider intelligence of people with DS more malleable than did non-professionals, which may seem counterintuitive given that the enhancement of the skills is the core of their mission.

It is worth noting that the professionals were more positive in their judgments: compared to non-professionals, they considered people with DS to be more educable, more intelligent and less stupid. Both groups expressed negative implicit attitude (the IAT effect) but professionals had a lower IAT score. We also found that implicit theory of the intelligence of people with DS predicted explicit judgments. The more participants (both professionals and non-professionals) endorsed entity theory for people with DS, the less they judged these people intelligent, and the more they judged them stupid. Implicit theory of intelligence considering people with DS did not predict neither the trait “educable” nor negative implicit attitudes in professionals. Overall, implicit theories of intelligence predicted a larger percentage of variance of judgments in the non-professionals than in the professionals.

Our results are in line with data reporting that considering intelligence as less malleable contributes to a more "entitative" perception of the group under consideration and then to more stereotypical judgments [29]. It is interesting to note that we showed a difference in the relationships between implicit theories of intelligence and judgments between professionals and non-professionals, suggesting that the experience of working with people with DS(thus providing relevant knowledge of these people) may offset the effects of conceptions of intelligence on judgments, in particular for educability. Social stereotyping of people with DS is more positive than for other people with intellectual disabilities [38], but judgments are still more negative than they are for typical people [30]. According to Plaks, Stroessner, Dweck and Sherman, stereotypical judgments are stronger with entity theorists [39]. Assessing participants looking at faces of people with DS, we found that the more they believed in entity theory, the more negative, and less positive, the judgments they expressed. Consequently, even though positive judgments are usually expressed overtly, we were able to confirm the negative aspect of the social stereotyping of DS, and this was consistent with the negative associations observed at the implicit level. Our research findings should improve the understanding of the social perception of people with intellectual disabilities. Not only are these people judged stereotypically, but their intelligence is also perceived as being different: it is seen as less malleable when compared to the intelligence of people, and even by professionals working in the field of intellectually disability. Enea-Drapeau, Huguet and Carlier showed that the level of intelligence of children with DS, when judged on the basis of facial features, was found to be misleading, notably in non-professionals [40]. All these findings confirm that there is a high risk that individuals can be prejudiced in their view of the intelligence of people with DS. When we consider what has been established in research in the general field of education with reports on the negative impact of fixed implicit theory of intelligence on teaching practices and school outcomes, the importance of our data can be seen. Our findings may pave the way to improve social interactions and educational practices in the field of intellectual disability. It has been shown that by inducing an incremental theory (growth mindset) in teachers and students, school outcomes, teaching and learning strategies can improve [18,41–44]. It has been established that implicit theories can influence teaching practices, teachers’ judgments and students’ learning strategies. Our study is a first step investigating implicit theories in the domain of people with intellectual disabilities. Investigating their effects on teaching and learning processes would be the logical next step.

Further studies should include an investigation into beliefs of people with intellectual disabilities about their own abilities and intelligence and the impact of these beliefs on learning processes and achievements bearing in mind that age educational variables can play an important role. Research has consistently demonstrated that a student’s belief in fixed intelligence will have negative effects on his/her goals, motivation, behavior, and self-esteem [12,45–47]. Pretzlik, Olsson, Nabuco and Cruz observed that pupils tend to adopt their teachers’ conception of intelligence [48]. The study by Koestner, Aube, Ruttner and Breed is the only one that focused on beliefs of people with intellectual disabilities about their own ability [49]. Koestner et al. found that the children with intellectual disabilities were significantly less likely to endorse effort attributions for failure (i.e. to hold an incremental theory) than were children without intellectual disabilities [49]. They also found that when an incremental conception of abilities is induced the same positive effect can be had on children with and without intellectual disabilities, both groups choosing high levels of challenge and reporting great interest/enjoyment.

Finally, in our study, responses on the TISs were at the explicit level and therefore subject to social desirability biases. In particular, for people working in the field of intellectual disability, it is politically incorrect to declare that intelligence is fixed at birth. It is therefore important to investigate conceptions of intelligence held by professionals but at a level where they do not feel obliged to express socially desirable judgments, and they do not inhibitthe possibility of supporting entity theory of intelligence. In a recent study, Mascret, Roussel and Cury presented initial data on IAT testing of teachers’ implicit conceptions of intelligence [50]. They showed at the automatic or implicit level no preference associating “intelligence” and “modifiable.” Unfortunately, the study by Mascret et al. did not compare explicit and implicit measures of conceptions of intelligence.

The present study is limited by the relatively small number of professionals, which precluded any study of the influence of seniority or specializations (e.g. as teacher or physiotherapist). A next step needed is to investigate the development of implicit theories during the vocational training of professionals and throughout their professional career.

## Conclusions

Conception of intelligence concerning people with DS was found less incremental than conception of typical people’s intelligence both in professionals and non-professionals. In regards to the potential effects of holding a fixed theory on learning situations, our results should lead to further investigations and perspectives of interventions, both in people with intellectual disabilities and in professionals of this domain, at the explicit and the implicit levels.
